# Involvement of heparanase in the pathogenesis of acute kidney injury: nephroprotective effect of PG545

**DOI:** 10.18632/oncotarget.16573

**Published:** 2017-03-25

**Authors:** Zaid Abassi, Shadi Hamoud, Ahmad Hassan, Iyad Khamaysi, Omri Nativ, Samuel N. Heyman, Rabia Shekh Muhammad, Neta Ilan, Preeti Singh, Edward Hammond, Gianluigi Zaza, Antonio Lupo, Maurizio Onisto, Gloria Bellin, Valentina Masola, Israel Vlodavsky, Giovani Gambaro

**Affiliations:** ^1^ Department of Physiology, The Rappaport Faculty of Medicine, Technion, Haifa, Israel; ^2^ Department of Laboratory Medicine, Rambam Health Care Campus, Haifa, Israel; ^3^ Department of Internal Medicine E, Rambam Health Care Campus, Haifa, Israel; ^4^ Department of Internal Medicine A, Rambam Health Care Campus, Haifa, Israel; ^5^ Department of Gastroenterology, Rambam Health Care Campus, Haifa, Israel; ^6^ Department of Internal Medicine, Hebrew University-Hadassah Medical Center, Jerusalem, Israel; ^7^ Department of Cancer and Vascular Biology Research Center, The Rappaport Faculty of Medicine, Technion, Haifa, Israel; ^8^ Zucero Therapeutics, Brisbane, Queensland, Australia; ^9^ Department of Medicine, Renal Unit, Verona, Italy; ^10^ Department of Biomedical Sciences, University of Padova, Catholic University of the Sacred Heart, Roma, Italy; ^11^ Department of Medicine, Columbus-Gemelli Hospital, Catholic University of the Sacred Heart, Roma, Italy

**Keywords:** acute kidney injury, heparanase, ischemia, inflammation, PG545

## Abstract

Despite the high prevalence of acute kidney injury (AKI) and its association with increased morbidity and mortality, therapeutic approaches for AKI are disappointing. This is largely attributed to poor understanding of the pathogenesis of AKI. Heparanase, an endoglycosidase that cleaves heparan sulfate, is involved in extracellular matrix turnover, inflammation, kidney dysfunction, diabetes, fibrosis, angiogenesis and cancer progression. The current study examined the involvement of heparanase in the pathogenesis of ischemic reperfusion (I/R) AKI in a mouse model and the protective effect of PG545, a potent heparanase inhibitor. I/R induced tubular damage and elevation in serum creatinine and blood urea nitrogen to a higher extent in heparanase over-expressing transgenic mice *vs*. wild type mice. Moreover, TGF-β, vimentin, fibronectin and α-smooth muscle actin, biomarkers of fibrosis, and TNFα, IL6 and endothelin-1, biomarkers of inflammation, were upregulated in I/R induced AKI, primarily in heparanase transgenic mice, suggesting an adverse role of heparanase in the pathogenesis of AKI. Remarkably, pretreatment of mice with PG545 abolished kidney dysfunction and the up-regulation of heparanase, pro-inflammatory (i.e., IL-6) and pro-fibrotic (i.e., TGF-β) genes induced by I/R. The present study provides new insights into the involvement of heparanase in the pathogenesis of ischemic AKI. Our results demonstrate that heparanase plays a deleterious role in the development of renal injury and kidney dysfunction, attesting heparanase inhibition as a promising therapeutic approach for AKI.

## INTRODUCTION

Acute kidney injury (AKI) is a common clinical disorder affecting 2-7% of hospitalized patients and some 20-50% of critically ill subjects [1, 2]. Renal ischemia and hypoxia, toxic insult and sepsis are the leading causes of clinical AKI [1, 2]. Specifically, AKI is characterized by decreased oxygen delivery, depletion of cellular energy stores and accumulation of toxic metabolites. The cellular depletion of ATP, a hallmark of ischemic injury, leads to a series of morphological, biochemical and physiological derangements [2–4]. Reperfusion of ischemic tissue, although necessary for repair, has been shown to exacerbate AKI owing to the generation of reactive oxygen and nitrogen species [2–6]. Despite the advances in critical care medicine, AKI is still associated with high morbidity and mortality assumedly due to delayed detection of the disease and lack of effective treatment [7].

Several strategies were proposed to deal with AKI [2, 3, 8]. Among these are antioxidants including acetylcysteine, antioxidant enzyme mimetics, erythropoietin, peroxisome-proliferator-activated receptor agonists, inhibitors of poly(ADP-ribose) polymerase, carbon monoxide-releasing molecules and other measures to enhance hypoxia inducible factors, statins, and adenosine [2, 3, 6]. Unfortunately, the clinical efficacy of these therapeutic approaches is inconsistent and disappointing. Therefore, there is a great need to improve our understanding of the pathogenesis of AKI and design new strategies to prevent kidney damage during AKI.

Heparan sulfate (HS) proteoglycans (HSPGs) are ubiquitous macromolecules associated with the cell surface and extracellular matrix (ECM) of a wide range of tissues including the kidney [9]. The HS chains bind to and assemble ECM proteins, thus playing important roles in ECM integrity, barrier function, and cell-ECM interactions [9]. HSPGs not only provide a storage depot for heparin binding molecules (i.e., growth factors, chemokines, enzymes) in the tissue microenvironment, but also regulate their accessibility, function and mode of action. It is therefore not surprising that a HS degrading enzyme, heparanase, is critically involved in pathological processes, including tumor growth, metastasis, angiogenesis, thrombosis, fibrosis, inflammation, autoimmunity and kidney dysfunction [10–18].

Emerging evidence suggests the involvement of heparanase in diabetic and nondiabetic proteinuric kidney diseases [19, 20]. For example, heparanase expression was shown to be upregulated in a number of animal models of renal diseases, including passive Heymann nephritis [21], puromycin aminonucleoside nephrosis (PAN) [22], adriamycin nephropathy (ADR-N) [23, 24], anti-glomerular basement membrane (GBM) nephritis [25], and diabetic nephropathy [26]; and in glomerular epithelial and endothelial cells cultured in ambient high glucose concentration [27]. As expected, heparanase upregulation was associated with reduced HS size in the GBM [20, 28]. Likewise, increased heparanase activity was detected in urine samples from diabetic patients with microalbuminuria [20, 27, 29–31], nondiabetic nephrotic syndrome, chronic kidney diseases (CKD) and kidney transplanted patients [29]. Notably, neutralization of heparanase activity, using either a sulfated oligosaccharide inhibitor (PI-88) or anti-heparanase antibodies, resulted in reduced proteinuria in ADR-N [32]. Similarly, Gil and colleagues [33] demonstrated that heparanase null mice fail to develop albuminuria and renal damage in response to streptozotocin-induced diabetes mellitus. Furthermore, a lower degree of albuminuria was detected in type 1 and type 2 diabetic mice treated with a heparanase inhibitor (SST0001 = Roneparstat) *vs*. mice treated with vehicle alone [33].

Although heparanase is recognized for its important role in diabetic nephropathy and other progressive CKD [34], its direct involvement and mode of action in the pathogenesis of AKI has not been studied thoroughly. We have previously reported that heparanase is involved in the onset and development of I/R-induced epithelial to mesenchymal transition (EMT) both *in vitro* and *in vivo* [35] and Lygizos et al [36] found that glomerular heparanase is activated during sepsis and contributes to septic AKI. Elucidation of the mechanism underlying the function of heparanase in AKI is critically important for the proper design of novel therapeutics approaches, including heparanase inhibitors such as PG545, Roneparstat and other compounds currently under intensive development and clinical testing [17, 37]. PG545, a synthetic fully sulfated HS mimetic with potent, long acting heparanase inhibiting capacity [38, 39], ended phase I clinical trial in cancer patients. The availability of PG545 and of heparanase over-expressing transgenic mice (Hpa-tg) [40] provides a most appropriate experimental platform to elucidate the involvement of heparanase in the pathogenesis of AKI. We report that PG545 abolished kidney dysfunction and the up-regulation of heparanase, pro-inflammatory (i.e., IL-6) and pro-fibrotic (i.e., TGF-β) mediators induced by I/R AKI.

## RESULTS

### Acute ischemic injury up-regulates renal heparanase expression and enzymatic activity

**Figure 1:**
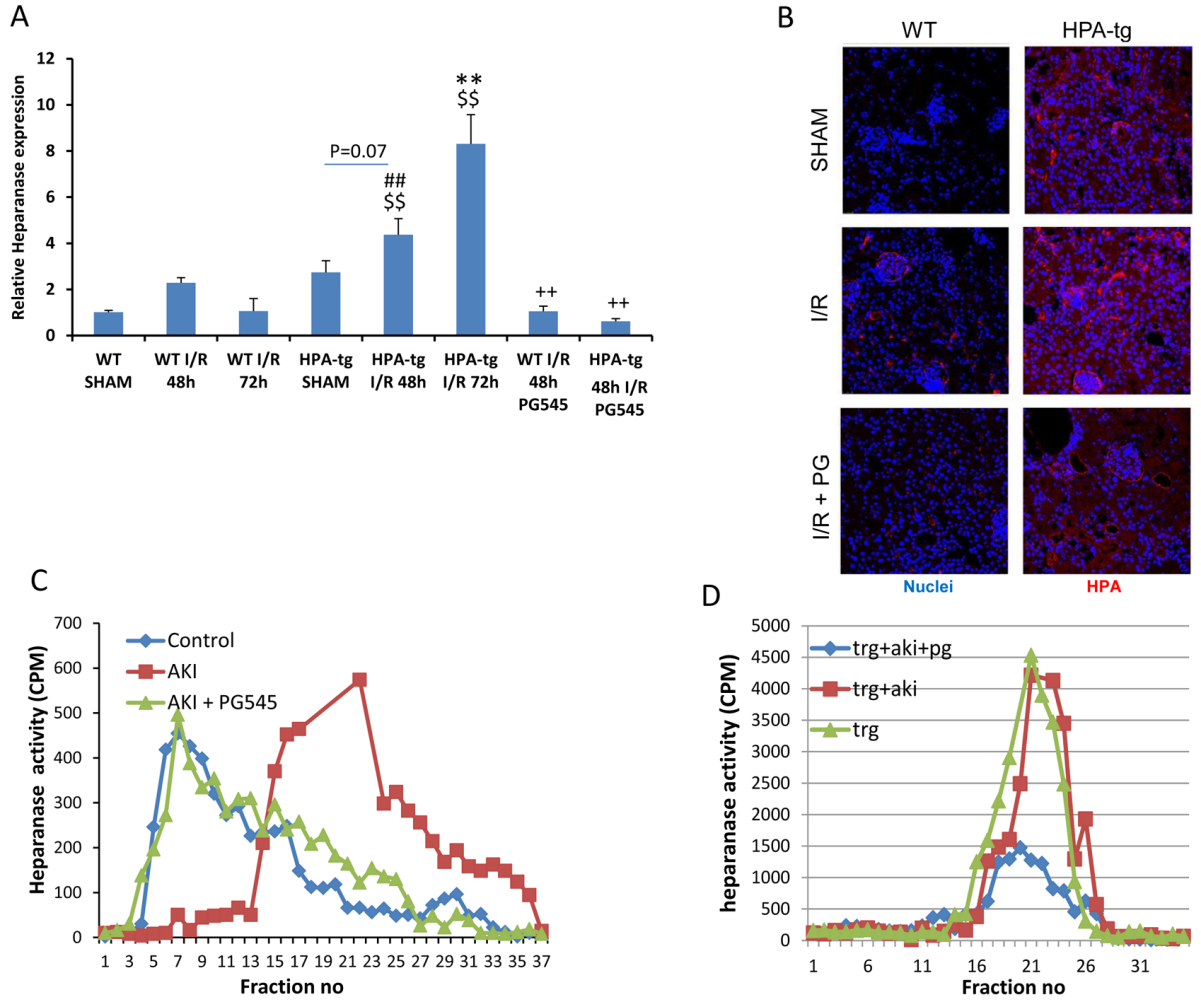
Heparanase regulation by I/R kidney injury **A**. Bar plot showing relative gene expression of HPSE evaluated by real-time PCR in renal tissue extract from *wt* and Hpa-tg mice untreated or pre-treated with PG545. Results were normalized to GAPDH expression. **B**. Representative immunofluorescence staining of heparanase in cortical renal tissues of *wt* and Hpa-tg mice 48 h after I/R kidney injury, with or without pre-treatment with PG545. Magnification 40X. Representative heparanase activity in the renal tissue of *wt* **C**. and Hpa-tg **D**. mice prior and post I/R in the presence or absence of PG545 pretreatment. I/R, ischemia/reperfusion. *p < 0.05, **p < 0.01 *vs*. corresponding sham; #*p* < 0.05, ##*p* < 0.01 *vs*. corresponding group w/o I/R ; $*p* < 0.05, $$*p* < 0.01 *vs*. corresponding wt; +*p* < 0.05, ++*p* < 0.01 *vs*. untreated I/R corresponding group.

Acute ischemic injury was induced by clamping both renal arteries for 30 minutes. As previously described [35], real-time PCR analyses (Figure 1A) confirmed an increment of heparanase expression in renal tissue of *wt* mice 48 h after ischemia and a more pronounced effect was noted in Hpa-tg mice at 72 h. Immunofluorescence staining of renal tissue of *wt* animals confirmed that acute ischemic renal injury up-regulated heparanase in glomeruli, tubular cells and interstitial cells (Figure 1B). As demonstrated in Figure 1C, heparanase enzymatic activity was markedly enhanced following AKI in *wt* mice. Thus, when incubated with sulfate-labeled ECM, extracts from kidney of *wt* mice that were subjected to AKI released high amounts of HS degradation fragments (peak at fraction 23) as compared with sham control mice. As expected, kidney tissue from untreated Hpa-tg mice exhibited high basal heparanase activity (Figure 1D). These results are supported by qPCR analysis (Figure 1A) and immunostaining (Figure 1B) showing that heparanase expression and immunoreactivity were increased following AKI induction in both *wt* and Hpa-tg mice. Importantly, when *wt* and Hpa-tg mice were pretreated with PG545, heparanase gene expression (Figure 1A), immunoreactivity (Figure 1B) and enzymatic activity (Figure 1C, 1D) were profoundly suppressed, signifying the remarkable protective effect of PG545 against I/R.

### Heparanase induces acute ischemic injury

#### Histology

**Figure 2:**
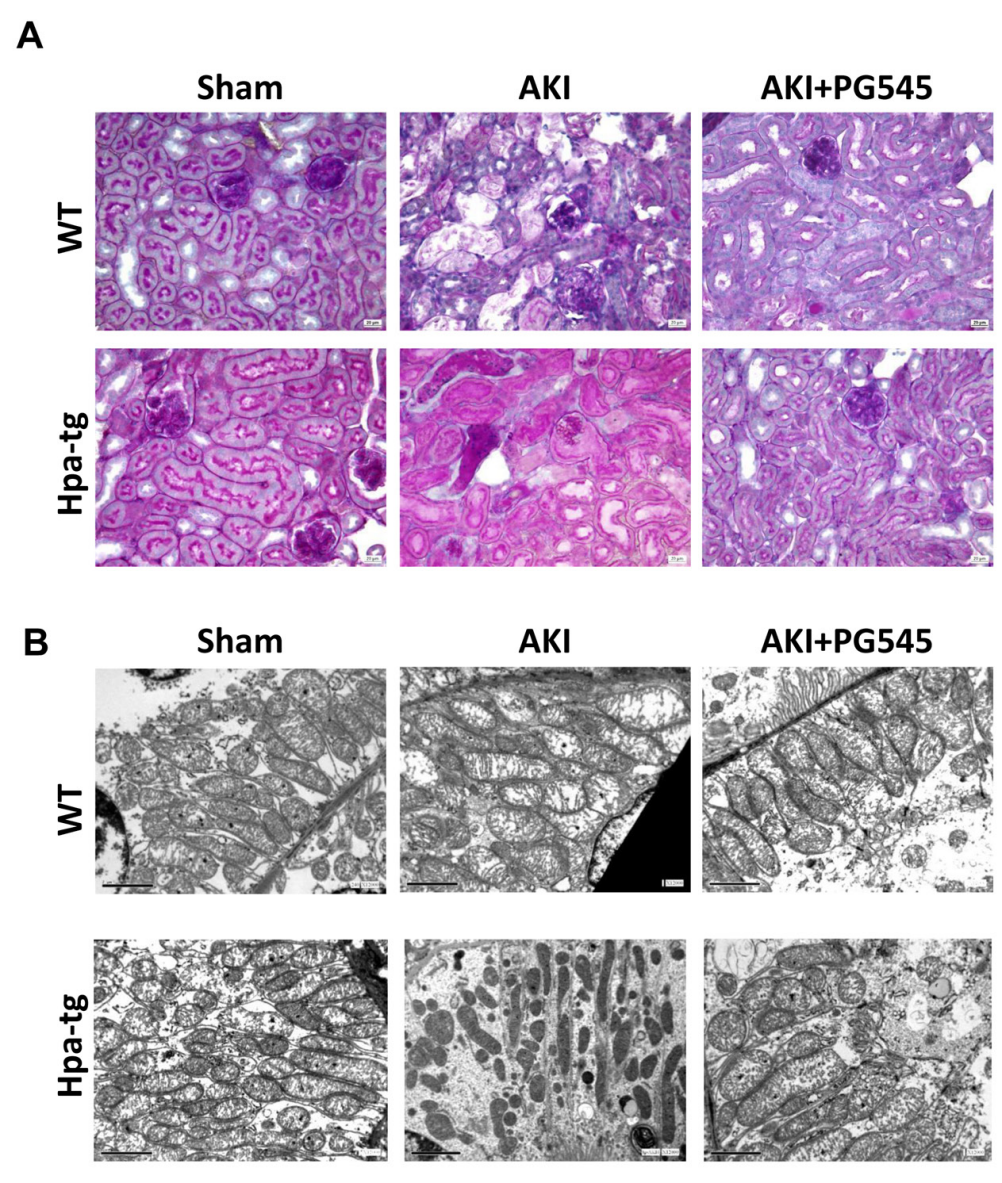
Ischemia/reperfusion (I/R) kidney injury in *wt* and Hpa-tg mice I/R kidney injury was induced in *wt* and Hpa-tg mice by 30 minutes of clamping of both renal arteries. Mice were sacrificed after 48 hours. **A**. Representative images of PAS staining of paraffin-embedded cortex sections from various experimental groups. Magnification 40x. **B**. Electron microscopy micrographs of cortical renal tissue from *wt* and Hpa-tg mice that were subjected to 30 minutes of clamping of both renal arteries in the absence or presence of PG545 (0.4 mg/mouse, i.v). Note mitochondrial swelling and damage to mitochondrial cristae. Note normal ultrastructural appearance of mitochondria from mice treated with PG545. Magnification 12,000×.

The structural changes in the kidney tissue of control and I/R *wt* and Hpa-tg mice were evaluated by PAS staining. We confirmed that at 48 h after I/R, *wt* mice showed acute tubular necrosis which included tubular lysis, loss of brush border and sloughed debris in the tubular lumen spaces (Figure 2A). While in *wt* mice the damage was partially attenuated after 72 h, the injury in Hpa-tg mice was more profound and persistent also after 72 h (not shown). In Hpa-tg mice there was a significant alteration in glomeruli and tubular structures. In particular, in Hpa-tg mice I/R produced a severe tubular damage with tubular dilatation, cell detachment from basement membrane, cast formation and loss of brush border (Figure 2A). Notably, these effects were partially prevented in response to pretreatment with PG545.

#### Ultrastructure alterations

Electron microscopy analyses of the renal tissue from the various experimental groups are presented in Figure 2B. As expected, induction of AKI resulted in remarkable mitochondrial alterations. Specifically, the mitochondria in the tubular cells of control *wt* and Hpa-tg mice exhibited elongated cylindrical shape, whereas induction of AKI in both *wt* and Hpa-tg mice resulted in fragmented mitochondria and transformation from filamentous shape into short rods (Figure 2B). These deleterious alterations were more profound in Hpa-tg mice as was evident by spherical shape and matrix vacuoles (cristolysis). Pretreatment with PG545 of either *wt* or Hpa-tg mice partially restored the tubular mitochondrial morphological changes induced by AKI (Figure 2B) as was evident by nearly normal ultrastructural appearance of the mitochondrial cristae (Figure 2B).

#### Kidney function

**Figure 3:**
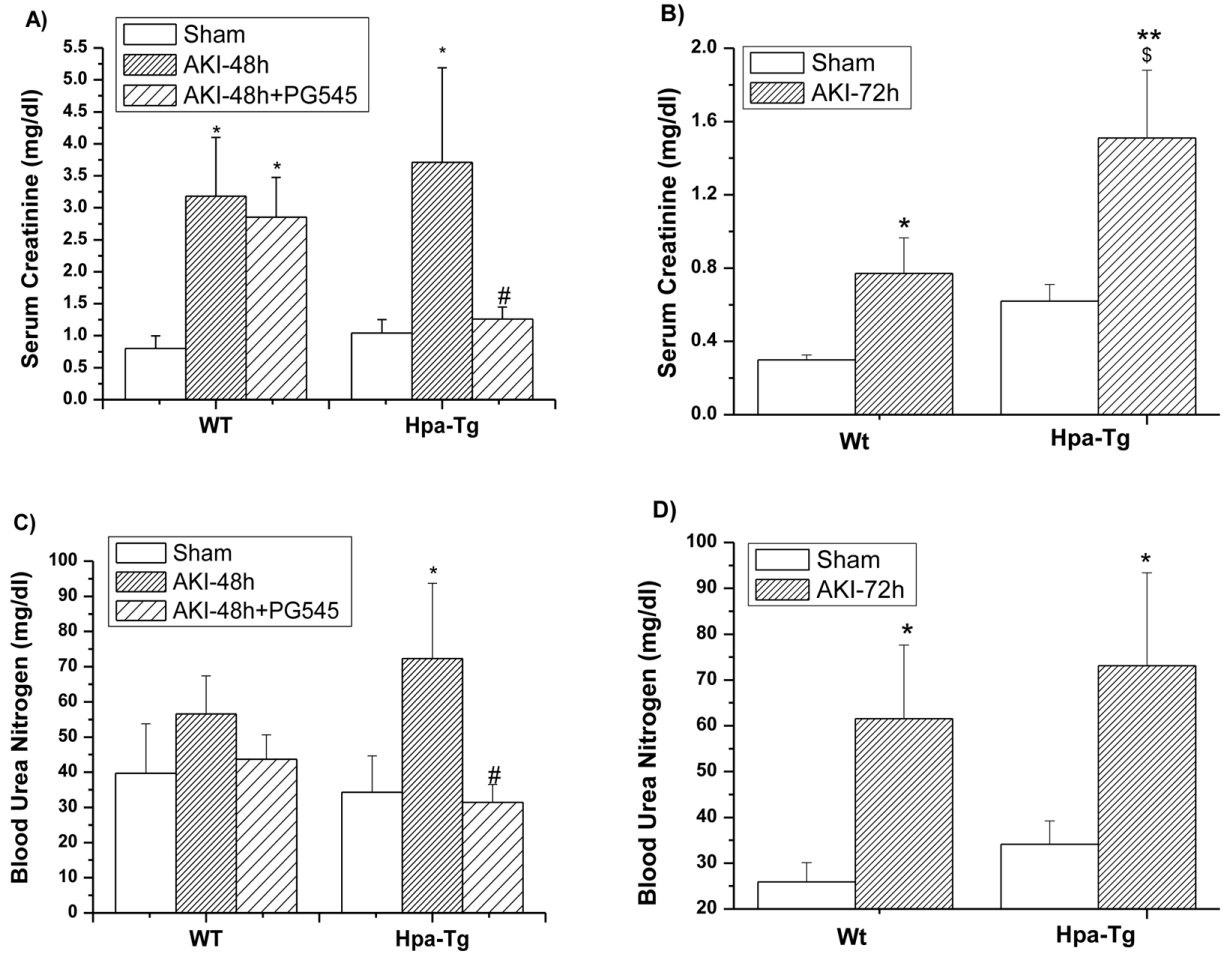
Biomarkers of kidney function in *wt* and Hpa-Tg mice after I/R Effect of AKI on serum creatinine (SCr) **A**., **B**. and Blood urea nitrogen (BUN) **C**., **D**. 48 h **A**., **C**. and 72 h **B**., **D**. after I/R insult in *wt* and Hpa-tg mice. Note that AKI induced a more profound increase in both SCr and BUN in Hpa-tg mice as compared with *wt* mice. Additional two groups of *wt* and Hpa-tg mice were pretreated one day prior to AKI induction with PG545 (0.4 mg/mouse, ip) and sacrificed 48 h after renal injury **A**., **C**. *p < 0.05, **p < 0.01 *vs*. sham; #*p* < 0.01 *vs*. untreated mice; $ *p* < 0.05 *vs*. *wt*.

Besides the histological changes, we further determined kidney function by measuring blood urea nitrogen (BUN) and serum creatinine (SCr) in the studied experimental groups (Figure 3). Induction of AKI in both *wt* and Hpa-tg mice was characterized by significant elevation of serum creatinine (SCr) (Figure 3A, 3B) and blood urea nitrogen (BUN) (Figure 3C, 3D), which was evident after 48 h and 72 h in both the *wt* and Hpa-tg mice (Figure 3), but was more prominent in the Hpa-tg mice after 72 h (Figure 3B, 3D). Pretreatment with PG545 attenuated the elevation of SCr and BUN in the Hpa-tg mice (Figure 3A, 3C).

### Heparanase inhibition attenuates epithelial mesenchymal transition

**Figure 4:**
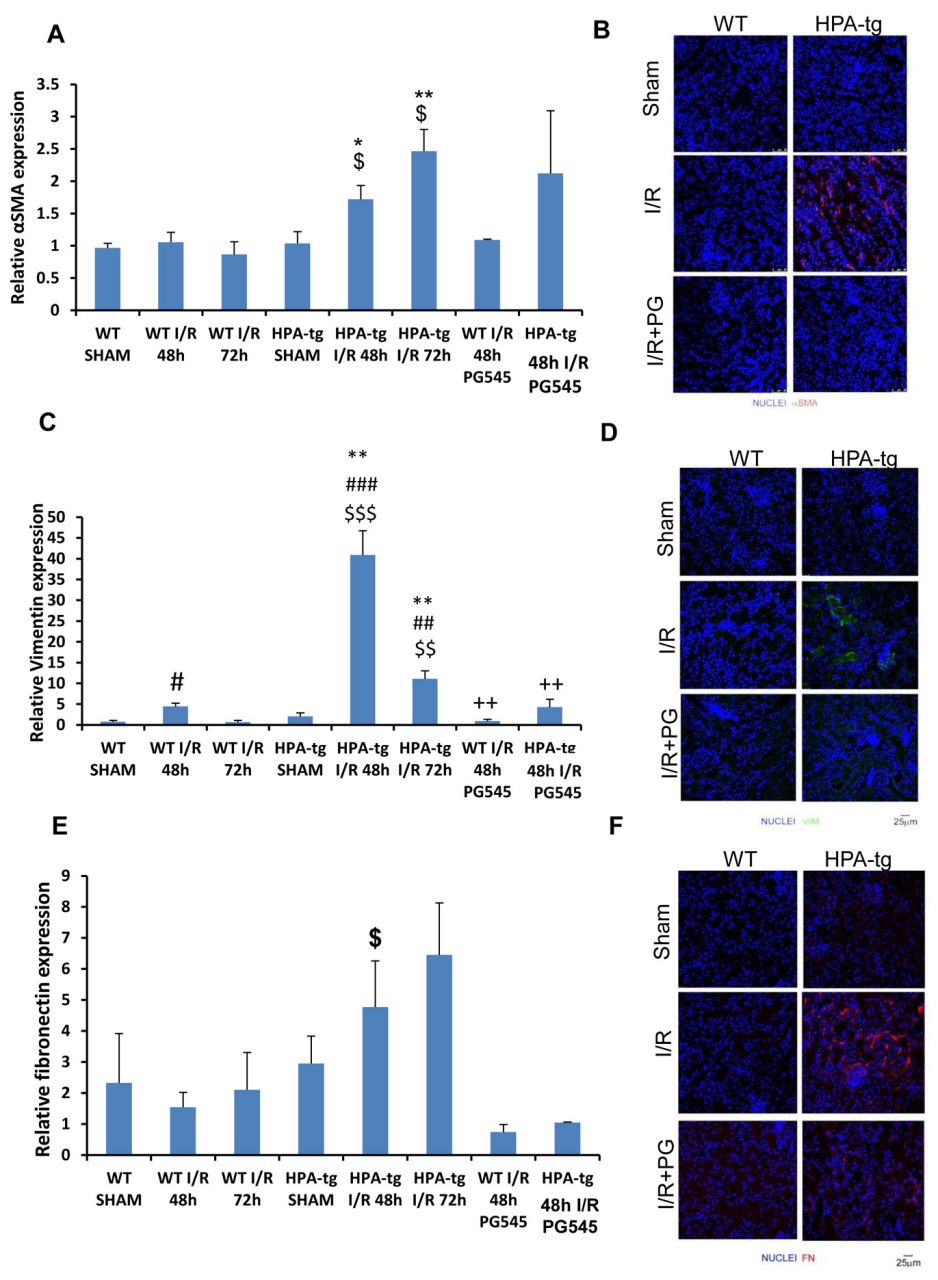
Expression of epithelial mesenchymal transition (EMT) markers in *wt vs* Hpa-tg mice after I/R kidney injury. Relative gene expression of **A**. α-SMA, **C**. VIM, and **E**. FN, were evaluated by real-time PCR in renal tissue extracts from *wt* and Hpa-tg mice that underwent I/R kidney injury with or without pre-treatment with PG545. Results were normalized to GAPDH expression. **p < 0.001 *vs*. *wt* SHAM mice. I/R, ischemia/reperfusion. Representative immunofluorescence staining of α-SMA **B**., VIM **D**. and FN **F**. in renal tissue of *wt* and Hpa-tg mice that underwent 48 h of I/R kidney injury with or without pre-treatment with PG545. Magnification 40x. *p < 0.05, **p < 0.01 *vs*. corresponding sham; #*p* < 0.05, ##*p* < 0.01 *vs*. corresponding group w/o I/R; $*p* < 0.05, $$*p* < 0.01 *vs*. corresponding wt; +*p* < 0.05, ++*p* < 0.01 *vs*. untreated I/R corresponding group.

Gene expression analysis of total kidney lysates and immunofluorescence staining for α-SMA (Figure 4A, 4B), vimentin (Figure 4C, 4D) and fibronectin (Figure 4E, 4F) showed a slight or no up-regulation following acute ischemic renal insult in *wt* mice. In contrast, Hpa-tg mice displayed a significant and early activation of these parameters. Specifically, while in *wt* mice α-SMA (Figure 4A) and vimentin (Figure 4C) expression in the kidney were slightly increased 48 h after acute renal ischemia and returned to basal levels after 72 h, Hpa-tg mice exhibited remarkable up regulation of SMA (Figure 4A), vimentin (Figure 4C) and FN (Figure 4E), both at 48 and 72 h. Similarly, we have previously reported the involvement of heparanase in epithelial mesenchymal transition, both *in vitro* and *in vivo* (35). Importantly, pretreatment with PG545 abolished the elevation in α-SMA, vimentin and fibronectin in the Hpa-tg mice (Figure 4A, 4C, 4E). Immunofluorescent staining confirmed the gene expression pattern on the protein level (Figure 4B, 4D, 4F), altogether emphasizing the involvement of heparanase in renal I/R and the associated epithelial mesenchymal transition.

### Heparanase affects TGF-β, ET-1, IL6, TNFα and cathepsin L expression

**Figure 5:**
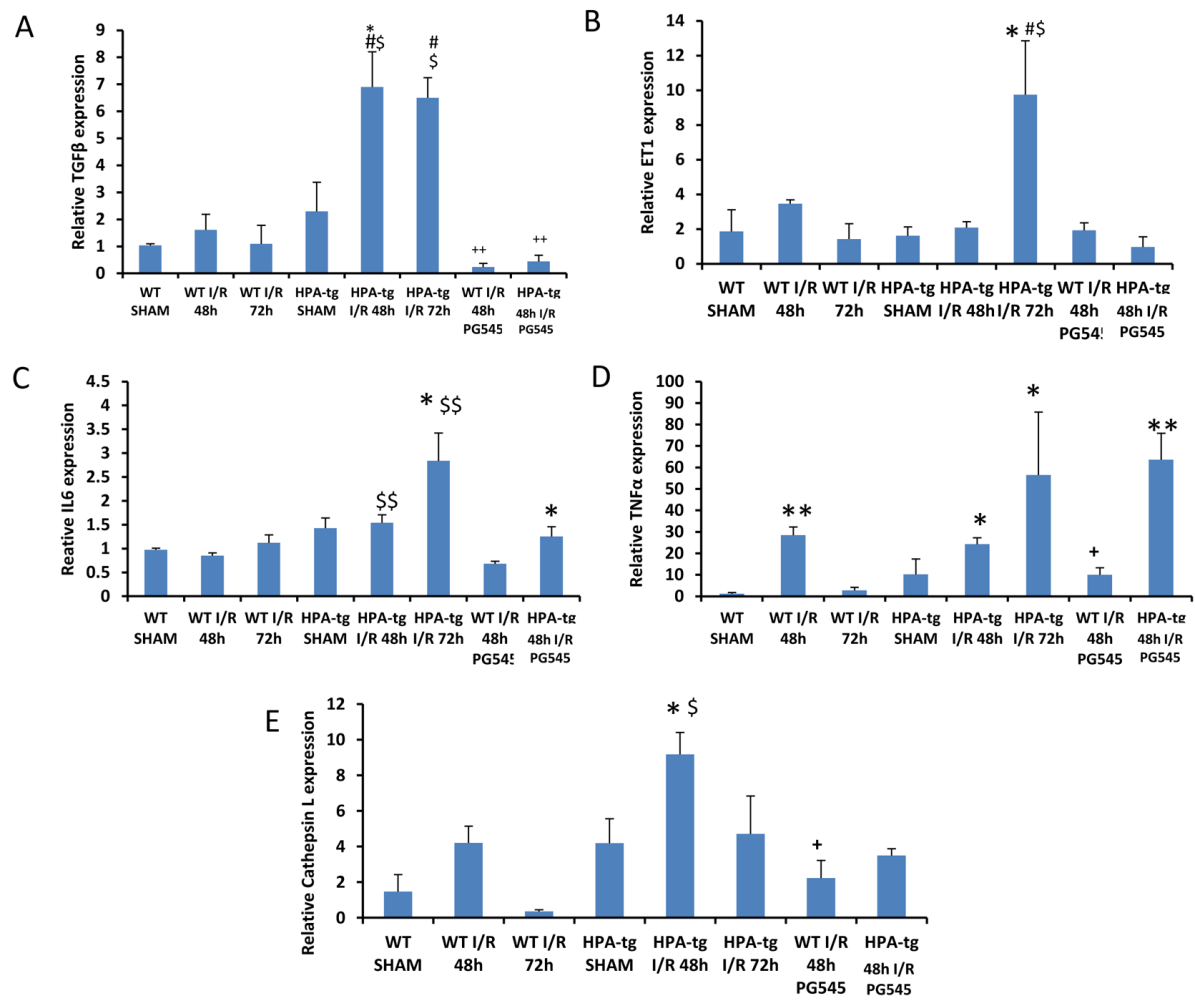
TGF-β, ET-1, IL6, TNFα and cathepsin L gene expression in *wt vs* Hpa-tg mice after I/R kidney injury. Bar plots representing relative gene expression of TGF-β **A**., ET-1**B**., IL6 **C**., TNFα **D**. and Cathepsin L **E**., evaluated by real-time PCR in renal tissue extracts from *wt* and Hpa-tg mice that underwent I/R kidney injury with or without pre-treatment with PG545. Results were normalized to GAPDH expression. *p < 0.05, **p < 0.001 *vs*. *wt* sham; #*p* < 0.05, ##*p* < 0.01 *vs*. corresponding group w/o I/R ; $*p* < 0.05, $$*p* < 0.01 *vs*. corresponding wt; +*p* < 0.05, ++*p* < 0.01 *vs*. untreated I/R corresponding group.

In *wt* mice, 48 and 72 h after ischemia, TGF-β mRNA levels were similar to sham operated animals. In contrast, in Hpa-tg mice, acute I/R injury induced significant up-regulation of the TGF-β gene at both 48 h and 72 h (Figure 5A). A similar pattern of ET-1 (Figure 5B) and IL6 (Figure 5C) upregulation was observed in Hpa-tg, but not *wt* mice that were subject to AKI, yet the upregulation of these genes was significant only at 72 h. Induction of AKI in *wt* and Hpa-tg mice was also associated with enhanced expression of TNFα (Figure 5D) and cathepsin L (Figure 5E), a key enzyme in the processing and activation of latent heparanase [41]. Again, the increased expression of these two genes was more profound in Hpa-tg mice. Remarkably, pretreatment with PG545 abolished the elevation in TGFβ, and to a lesser extent the upregulation of ET-1, IL-6, cathepsin L and TNFα. Altogether, it appears that biomarkers of fibrosis, EMT and inflammation were upregulated in I/R induced AKI, primarily in Hpa-tg mice, and this elevation was prevented to a large extent following pretreatment with PG545.

## DISCUSSION

Despite the high prevalence of AKI and its association with an alarming increase in morbidity and mortality, the therapeutic approaches for AKI are still disappointing and rely mainly on supportive measures. This deficiency stems mainly from the poor understanding of the pathogenesis of AKI. The current research is therefore of special significance since it provides new insights into the mechanism underlying ischemic AKI, a major etiology of AKI. Specifically, we demonstrated that I/R of the kidney induced renal injury in both *wt* and Hpa-tg mice as was evident by typical tubular damage including cell lysis, loss of brush border and sloughed debris in tubular lumen, which was more profound in Hpa-tg mice (Figure 2). The latter also displayed exaggerated elevation in SCr and BUN. Moreover, TGF-β, fibronectin, vimentin and α-smooth muscle actin, biomarkers of epithelial mesenchymal transition [35, 42], were upregulated in I/R induced AKI, especially in Hpa-tg mice, indicating an adverse role of heparanase in the pathogenesis AKI. This notion is further supported by the fact that pretreatment with PG545, a potent long acting inhibitor of heparanase [38], abolished the adverse renal changes induced by I/R and improved kidney function. Collectively, these findings clearly implicate heparanase in the pathophysiology of AKI, and suggest heparanase inhibitors as preventive/therapeutic option for this common clinical setting. Given that transgenic mice may differ from their *wt* counterparts in various aspects, each experiment was performed with both *wt* and Hpa-tg mice, yielding the same results (i.e., upregulation of biomarkers of fibrosis, inflammation and EMT and down regulation/protection by the heparanase inhibitor PG545), albeit to a lower extent in *wt* mice. Overall it appears that the disease model is operating in *wt* mice in a manner similar to that seen in Hpa-tg mice.

Despite the remarkable advances in nephrology, the pathophysiology of AKI remains largely obscured. Thus, research involving genetically modified animal models is a critical step in providing a new perspective into the pathophysiology of AKI. Indeed, the Hpa-tg mouse model [40] provides an appropriate experimental platform to elucidate the involvement of heparanase in the pathogenesis of AKI. Of note, Hpa-tg mice revealed increased levels of urinary creatinine at baseline, indicating a slight effect of heparanase on renal function even without induction of AKI (40), although this increase did not reach statistical significance as compared with *wt* mice (Figure 3). This result further supports the involvement of heparanase in kidney dysfunction, showing that the heparanase level in Hpa-tg mice is by itself not enough to induce acute damage, whereas its combination with I/R results in AKI to a higher extent in Hpa-tg than in wild type mice. Similar to other heparanase-inhibiting heparin-like compounds PG545 displays some anti-coagulant properties, but this side effect was not detected in the AKI model nor in numerous tumor models in which mice are treated with PG545 for weeks [38]. Also, PG545 was found safe in phase I clinical trial in cancer patients [17, 37]. Our preliminary studies indicate that administration of PG545 post I/R failed to restore kidney damage and function. Yet, our pre-treatment approach maybe of clinical relevance in cases where AKI may occur (i.e., patients who undergo imaging involving i.v radiocontrast administration; patients undergoing major vascular surgery and cardiopulmonary bypass). So far there is no effective preventive treatment to minimize the incidence of AKI under these and other clinical settings, suggesting that PG545 may constitute a novel prophylactic therapeutic approach.

Unlike the Hpa-tg mice, results obtained with heparanase knockout mice [43] were not straightforward. AKI was induced by I/R also in the Hpa-KO mice, albeit to a lower extent than in *wt* and Hpa-tg mice, as revealed by the respective BUN and creatinine values. We have previously reported that the Hpa-KO mice reveal compensations for the lack of heparanase. These include up-regulation of matrix degrading enzymes and pro-inflammatory mediators [43], resulting, paradoxically, in several phenotypes that closely resemble phenotypes seen in the Hpa-tg mice, making it difficult to interpretate data obtained with the Hpa-KO mice.

The availability of PG545 allows exploring potential therapeutic effects of heparanase inhibition in AKI. The latter is of special interest since in contrast to the well-established association between heparanase and glomerular diseases [20, 28], the role of heparanase in renal EMT associated with I/R injury has only recently been addressed [35]. Notably, a role for heparanase was identified in several proteinuric nephropathies [20, 23, 28], mainly in the pathogenesis of diabetic nephropathy [26, 33] and in a model of septic AKI [36]. The latter is not surprising in light of the emerging data linking heparanase and inflammatory responses, suggesting a role of this enzyme in AKI. Indeed, Lygizos et al [36] found that glomerular heparanase is activated during sepsis and contributes to septic AKI. Specifically, the authors induced polymicrobial sepsis in mice using cecal ligation and puncture (CLP) in the presence or absence of competitive heparanase inhibitors (heparin or nonanticoagulant N-desulfated re-N-acetylated heparin). CLP-treated mice demonstrated early activation of glomerular heparanase with coincident loss of glomerular function, as indicated by an increase in blood urea nitrogen (BUN) and decrease in GFR. Administration of heparanase inhibitors two hours prior to CLP attenuated the deleterious consequences of sepsis, suggesting that glomerular heparanase is active during sepsis and contributes to septic renal dysfunction *via* uncharacterized mechanisms. In line with these findings, bilateral renal ischemia/reperfusion (I/R) in syndecan-1 deficient mice resulted in increased initial renal failure and tubular injury compared with *wt* mice [44]. In additional study, these authors have shown that within 24 h after renal I/R, HSPGs expressed at the ab-luminal side of peri-tubular capillaries are induced to bind L-selectin and the monocyte chemoattractant protein-1, and that HSPGs facilitate monocyte extravasation [45]. A vicious circuit of heparanase-driven molecular events promoting chronic inflammation and renal injury has recently been described [11, 33]. This circuit is fueled by heterotypic interactions among glomerular, tubular, and immune cell compartments. It appears that latent heparanase, over-expressed by glomerular, epithelial and interstitial cells and post-translationally activated by cathepsin L, sustains continuous activation of kidney-damaging macrophages *via* degradation of HS and release of chemokines anchored within the ECM network and cell surfaces [11]. Moreover, active heparanase governs macrophage activation *via* HS degradation fragments which activate TLR2 and TLR4 [46], leading to increased levels of TNF-α thereby creating inflammatory conditions fostering macrophage-mediated renal injury and up-regulation of heparanase expression. This scenario is further supported by our findings. First, Hpa-tg mice were more susceptible to I/R-induced renal damage and kidney dysfunction as compared with their *wt* counterparts subjected to the same surgical maneuver. Second, using gene silencing approaches we further revealed an essential involvement of heparanase in the development of I/R-induced epithelial damage and suggested that its inhibition may signify a therapeutic approach to minimize/prevent ischemic-induced renal fibrosis [35, 47]. Both, our previous *in vitro* studies applying cultured epithelial cells that were subjected to I/R [35, 47] and the current *in vivo* ischemic AKI model in *wt* and *Hpa-tg* mice exhibited upregulation of heparanase along over-expression of pro-inflammatory and pro-fibrotic cytokines (i.e., TGF-β, IL-6, TNFα) as well as markers of EMT (i.e., VIM, FN, αSMA). Activated macrophages secrete TNFα and cathepsin L, which stimulate further production and activation of heparanase by these cells and by ischemia-induced renal injured cells.

Upregulation of the TGF-β gene suggests that I/R triggers renal fibrogenesis, most likely through epithelial-mesenchimal transition (EMT) of tubular cells [48]. Hparanase seems to play a major role in these processes as was evident by the exaggerated overexpression of TGF-β in Hpa-tg mice exposed to I/R. While the critical causal involvement of TGF-β in EMT and tissue fibrosis is well documented [42, 49], little is known about a crosstalk between heparanase and TGF-β [48]. Even more so, a crosstalk between heparanase and ET-1 [50, 51] in AKI has not been reported. Evidence for the combined involvement of heparanase, TGF-β and ET-1 in the fibrosis/inflammatory response to AKI is provided by showing that treatment with PG545 prior to I/R maintained a normal epithelial phenotype and abolished the EMT transcriptional program exemplified by a marked decrease in the levels of VIM, FN, α-SMA, IL-6, ET-1 and TGF-β. PG545, a potent heparin-like inhibitor of heparanase enzymatic activity, markedly decreases the ability of heparanase to release and potentiate the activity of ECM-bound growth factors [37], further supporting PG545 as potential anti-fibrotic and kidney protective drug. Down-regulation of heparanase expression in response to PG545 was noted in 4T1 breast carcinoma model [52]. This effect was attributed in part to inhibition of growth factor-stimulated ERK activation by PG545 and the resulting inhibitory effect on the transcription factor early growth response 1 (EGR1), known to induce heparanase gene expression [53]. Given the multi target genes regulated by EGR1 [54], the same mechanism may explain the effect of PG545 on the expression TGF-β, TNF-α, fibronectin and possibly other cytokines. Similarly, we have recently reported that heparanase activates Erk, p38 and JNK signaling, leading to increased c-Fos levels and induction of cytokine expression [55]. Suppression of this cascade by PG545 may affect the transcription of multiple genes including heparanase.

One of the studied parameters that might be of special relevance to kidney damage is ET-1, a vasoconstrictor released by endothelial cells [56]. ET-1 acts *via* two receptors subtypes ETA and ETB to modulate vasoconstriction, proliferation, inflammation, ECM production, and fibrosis in various target organs including the kidney [57, 58]. An evidence for the deleterious role of ET-1 in renal damage is derived from the nephroprotective effects of ET-1 antagonists in experimental and clinical models of AKI [59]. Previous studies have demonstrated a linkage between ET-1 and heparanase [60]. Such an association is essential for the development of DN, as was proven by the observation that heparanase-deficient mice were protected against diabetes-induced proteinuria and renal damage [33]. Addition of ET-1 increased heparanase mRNA expression and enzymatic activity in cultured podocytes [60]. Our results clearly show a positive correlation between renal heparanase and ET-1. Both were upregulated during AKI and inhibition of heparanase was associated with a decline in ET-1, suggesting a causal relationship between ET-1 and heparanase during ischemic AKI.

In summary, our results indicate that heparanase is involved in the pathogenesis of ischemic AKI. Moreover, we demonstrated that pretreatment with a potent heparanase inhibitor (PG545) exerts nephroprotective effect against this clinical setting. Thus, our study provides a better perspective on the pathogenesis of AKI and the potential development of therapeutic/preventive interventions.

## MATERIALS AND METHODS

### Animal model

Studies were conducted on *wt* (Balb/c) and corresponding heparanase-overexpressing (Hpa-tg) mice weighing 23-30 gr and maintained on standard rat chow and water *ad libitum*. Mice were crossed for over 10 generations with Balb/c wt mice to produce pure genetic background. The Hpa-tg mice appeared normal and exhibited a normal life span and breeding behavior [40]. Western blot analysis revealed high expression of heparanase in the liver and kidney *vs*. low expression in the spleen of Hpa-tg *vs*. control mice [40, 61]. The study was performed according to the guidelines of the Animal Use and Care Committee, Technion (Haifa), and according to the Guide for the Care and Use of Laboratory Animals (NIH Publication no. 85-23, 1996) as approved by the local committee for supervision of animal experiments.

### Induction of renal ischemia

Acute ischemia was induced in *wt* (Balb/c) and Hpa-tg mice as described by Mishra et al [62]. *Wt* and Hpa-tg mice were anesthetized with sodium pentobarbital (50 mg/kg, IP) and placed on a controlled heating table, keeping the body temperature at 37°C. Both right and left renal arteries were exposed and clamped for 30 min during which time the kidney was kept warm and moist. The clamp was then removed, the kidney was observed for the return of blood flow, and the abdominal wall incision was sutured. Mice were allowed to recover in a warmed cage for 48 and 72 h and blood samples were obtained at each time point for measurement of creatinine and urea. At these time points, kidney dysfunction and renal damage peak, especially 48 h post I/R [63]. Additional groups of *wt* and Hpa-tg mice were pretreated one day prior to AKI induction with PG545 (0.4 mg/mouse, ip). The kidneys were harvested and weighted. One half of the left kidney was snap frozen in liquid nitrogen and stored at −70°C until further molecular processing; the other half was fixed in formalin, paraffin-embedded, and sectioned (4 μm). Paraffin sections were stained with hematoxylin-eosin and examined histologically. The right kidney was embedded in isopentane and frozen sections (4 μm) were obtained for immunohistochemistry [35]. Sham operated mice that underwent an identical procedure except renal artery clamping, served as controls. The number of mice per group (n) ranged from 6 to 12.

### Quantitative real-time PCR

Total RNA was extracted with TRIzol (Sigma) and RNA (1 µg) was amplified using one step PCR amplification kit, according to the manufacturer's (ABgene, Epsom, UK) instructions, essentially as described [55]. The moue primer sets used are listed in Table 1.

**Table 1:** Primers sequence list

Gene	Forward 5’-3’	Reverse 5’-3’
GAPDH	GGCAAATTCAACGGCACAGT	GTCTCGCTCCTGGAAGATGG
HPSE	CAAGAACAGCACCTACTCAAG	AGCAGTAGTCAAGGAGAAGC
α-SMA	TGCTGGACTCTGGAGATGGT	ACGAAGGAATAGCCACGCTC
VIM	TCCAGAGAGAGGAAGCCGAA	AAGGTCAAGACGTGCCAGAG
TGF-β	GTGTGGAGCAACATGTGGAACTCTA	CGCTGAATCGAAAGCCCTGTA
ET-1	TGCTGTTCGTGACTTTCC	TGTTGACCCAGATGATGTC
IL6	CTGCAAGAGACTTCCATCCAGTT	GAAGTAGGGAAGGCCGTGG
TNFa	CATCTTCTCAAAATTCGAGTGACAA	TGGGAGTAGACAAGGTACAACCC
Cathepsin L	GTGGACTGTTCTCACGCTCA	ATCCACGAACCCTGTGTCA

### Immunofluorescence

Sections (4 μm) were cut from paraffin embedded tissue and non-specific binding sites were saturated for 1 h at 37°C with PBS, 5% BSA. Sections were incubated at 4°C overnight with primary antibodies diluted in PBS supplemented with 1% BSA. The primary antibodies used were directed against vimentin (Santa Cruz sc-7557), fibronectin (Santa Cruz sc-9068), α-SMA (Sigma A5228), or heparanase (Santa Cruz sc-25826). Primary antibodies were visualized with anti-mouse AlexaFluor-488, anti-goat-FITC, anti-rabbit-Cy3, and anti-mouse-TR secondary antibodies. Cell nuclei were visualized by Hoechst 33258. Images were obtained by a confocal LeicaSP5 microscope [35].

### Heparanase activity

Preparation of Na_2_^35^SO_4_-labeled ECM-coated 35-mm dishes and determination of heparanase activity were performed essentially as described [55, 64]. Briefly, freshly collected renal tissues were homogenized and extracted in phosphate/citrate buffer (pH 5.2) by three cycles of freeze/thaw. Tissue lysates were incubated (overnight, 37°C) with ^35^S-labeled ECM. The incubation medium (1 ml) containing sulfate-labeled degradation fragments was subjected to gel filtration on a Sepharose CL-6B column. Fractions (0.2 ml) were eluted with PBS and their radioactivity was counted in a β-scintillation counter. Degradation fragments of HS side chains produced by heparanase are eluted at 0.5 < Kav < 0.8 (fractions 12-29). Nearly intact HSPGs released from the ECM are eluted just after the Vo (Kav < 0.2, fractions 3-10) [59, 60]. These high molecular weight products are released by proteases that cleave the HSPG core protein.

### Electron microscopy

Renal tissues from the various experimental groups were fixed in 3.5% glutaraldehyde and rinsed in 0.1 M sodium cacodylate buffer, pH 7.4. Tissue blocks (1 mm^3^) were post-fixed with 2% OsO_4_ in 0.2 M cacodylate buffer for 1 h, rinsed again in cacodylate buffer to remove excess osmium, immersed in saturated aqueous uranyl acetate, dehydrated in graded alcohol solutions, immersed in propylene oxide, and embedded in Epon 812. Ultrathin sections (80 nm) were mounted on 300-mesh, thin-bar copper grid, counterstained with saturated uranyl acetate and lead citrate. Sections were examined with a transmission electron microscope (Jeol 1011 JEM) at 80 KV.

### Chemical analyses

Blood urea nitrogen and serum creatinine levels were determined using commercially available colorimetric kits (Cayman, Ann Arbor, MI; Bioassay Systems, Hayward, CA, respectively).

### Statistical analysis

Mean ± SEM of the real-time PCR data were calculated with Rest2009 software. Gene expression differences in mouse samples were analyzed by linear regression models with group (WT-sham, WT-I/R 48h, WT-I/R 72h, TG-sham, TG-I/R 48 h, TG-I/R 72 h) entered as a categorical variable. Bonferroni-corrected adjusted means and differences were computed using the WT-sham as the referent group. A Bonferroni-corrected p-value < 0.05 was considered as statistically significant.
